# Making connections in the brain

**DOI:** 10.7554/eLife.32064

**Published:** 2017-10-26

**Authors:** Alex TL Leong, Ed X Wu

**Affiliations:** 1 Laboratory of Biomedical Imaging and Signal Processing, Department of Electrical and Electronic EngineeringUniversity of Hong KongHong Kong SARChina; 2 School of Biomedical Sciences, Li Ka Shing Faculty of MedicineUniversity of Hong KongHong Kong SARChina

**Keywords:** slow waves, BOLD fMRI, calcium recordings, resting-state functional connectivity, Rat

## Abstract

Simultaneous measurements of neuronal activity and fMRI signals in the rat brain have shed new light on the origins of resting-state fMRI connectivity networks.

**Related research article** Schwalm M, Schmid F, Wachsmuth L, Backhaus H, Kronfeld A, Aedo Jury F, Prouvot PH, Fois C, Albers F, van Alst T, Faber C, Stroh A. 2017. Cortex-wide BOLD fMRI activity reflects locally-recorded slow oscillation-associated calcium waves. *eLife* **6**:e27602. doi: 10.7554/eLife.27602

Functional MRI (fMRI) is a non-invasive technique that measures changes in the amount of oxygenated blood supplied to various regions of the brain, and this BOLD signal (short for blood-oxygen-level dependent signal) is used as a proxy for activity in these regions of the brain. fMRI measurements made when the brain is 'at rest' – that is, when the subject is not performing any specific task – have revealed the existence of long-range networks connecting different regions of the brain (Biswal et al., 1995; Fox and Raichle, 2007; Smith et al., 2013; Shen, 2015). Numerous studies have demonstrated that changes in these resting-state fMRI connectivity networks are involved in a range of cognitive functions (Lu et al., 2012; Raichle, 2015; Ash et al., 2016). However, despite the enormous potential of resting-state fMRI to explore many areas of neuroscience, the neural basis of these connectivity networks remains elusive.

Now, in eLife, Albrecht Stroh, Cornelius Faber and co-workers at institutions in Mainz, Frankfurt and Münster – including Miriam Schwalm, Florian Schmid and Lydia Wachsmuth as joint first authors – report new insights into the origins of resting-state fMRI connectivity (Schwalm et al., 2017). In brief, Schwalm et al. used resting-state fMRI to monitor whole-brain activity in rodents, while simultaneously imaging the activity of populations of neurons in the cortex via fluorescence signals from calcium ions (Ca^2+^). This made it possible to examine the relationship between resting-state fMRI signals and specific neurophysiological events.

Ca^2+^ measurements often reveal slow oscillations – rhythmic low-frequency waves generated by the rise and fall of neuronal activity in the cortex. Schwalm et al. showed that in rats anesthetized with isoflurane, the Ca^2+^ signals in the cortex displayed between about 8 and 20 large spontaneous peaks per minute. These peaks most likely reflect the synchronized firing of populations of neurons, otherwise known as the 'up' states of slow oscillations (Steriade et al., 1993).

To analyze their data, Schwalm et al. devised a new approach that involved classifying the peaks in Ca^2+^ activity as binary events. Then, using a statistical approach called the general linear model (GLM) method, they compared these binary Ca^2+^ events with the resting-state fMRI signals that were acquired simultaneously. This made it possible to identify resting-state fMRI connectivity networks that reflect the spatial extent of these Ca^2+^ events (Figure 1). This analysis revealed a connectivity network spanning the cortex, including the somatosensory and visual cortices, that correlated with the slow Ca^2+^ events.

**Figure 1.**
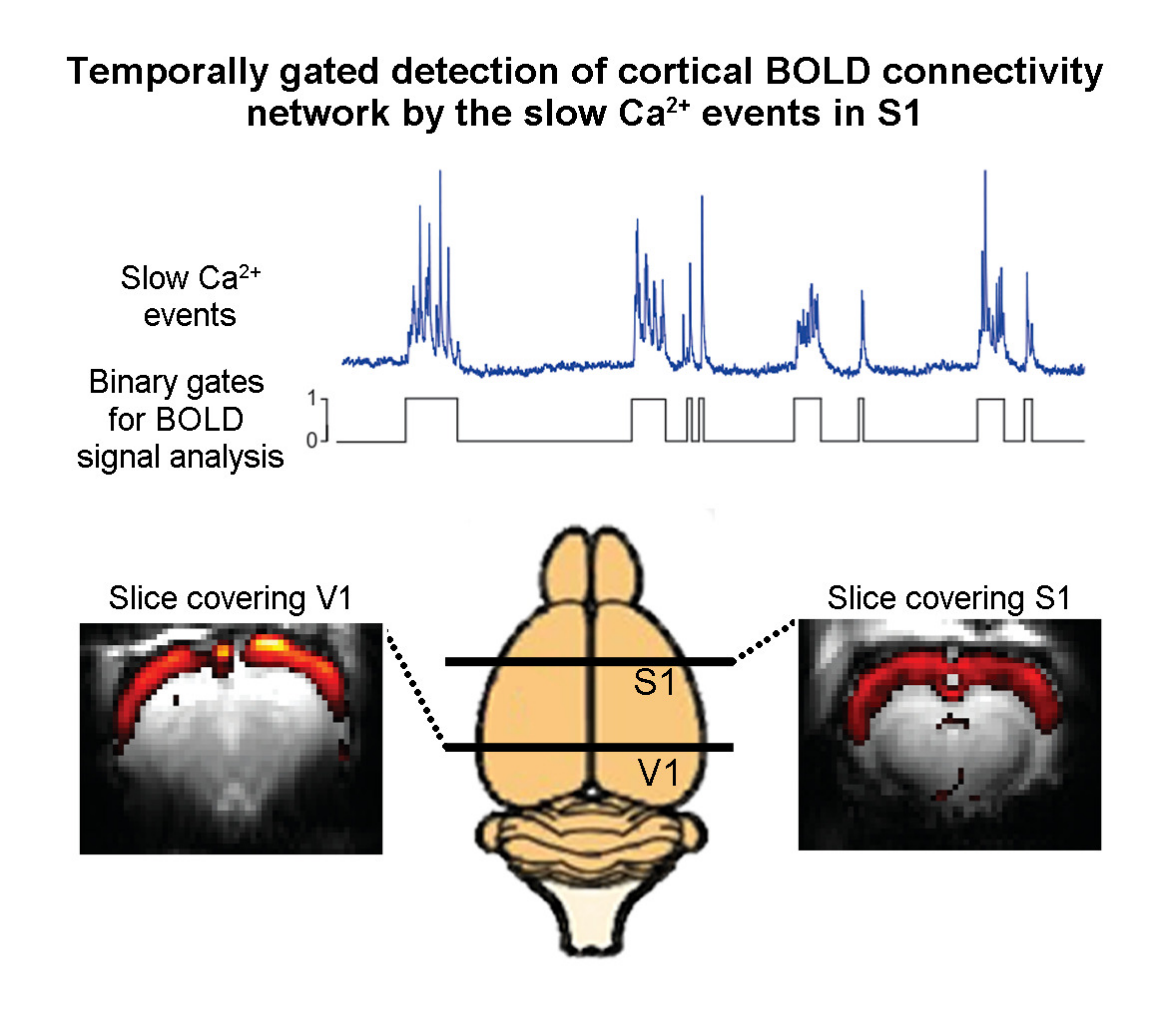
Combining resting-state functional MRI measurements of blood oxygenation and calcium recordings of spontaneous neural activity in the rat brain. The blue trace shows slow oscillations in a calcium recording of spontaneous neural activity in the primary somatosensory cortex; the trace shown here is approximately 80 seconds long. Schwalm et al. converted such traces into binary signals (black) and then used this binary signal to analyze the results of resting-state functional MRI measurements on the whole brain. This analysis revealed that the slow oscillations generate a resting-state fMRI connectivity network (red and yellow) that extends cortex-wide from the primary somatosensory cortex (S1) to the primary visual cortex (V1).

Since GLM-based methods are not commonly used in resting-state fMRI connectivity analysis, Schwalm et al. then confirmed this finding using more conventional approaches (such as independent component analysis and seed-based techniques). Moreover, they did not find any evidence for a cortex-wide connectivity network in rats that had been sedated with medetomidine (and which do not exhibit slow oscillations). These results indicate that resting-state fMRI connectivity phenomenon may be partially attributed to a defined neurophysiological event, namely the occurrence of slow oscillations.

The slow Ca^2+^ events seen in the cortex have certain characteristics in common with the infra-slow fluctuations in BOLD activity that signal the presence of resting-state fMRI connectivity networks. Typically, resting-state connectivity exhibits synchronized patterns of fluctuations in BOLD activity in both hemispheres (for example, in the bilateral sensory cortices). It was reported recently that Ca^2+^ events in the excitatory neurons of layers 2/3 and 5 of the bilateral sensory cortices coincide with the bilateral resting-state fMRI connectivity network (Ma et al., 2016). Further, another recent study revealed an additional Ca^2+^ event propagating globally across the cortex that coexists with the bilateral Ca^2+^ events (Matsui et al., 2016), suggesting the presence of an additional resting-state connectivity network. Here, Schwalm et al. revealed a resting-state fMRI connectivity correlate of such a global cortical Ca^2+^ event.

Slow oscillations or other forms of low-frequency neural activity have also been reported to be a key contributor to resting-state thalamo-cortical-thalamic networks (Crunelli and Hughes, 2010; Leong et al., 2016; Xiao et al., 2017) and hippocampal-cortical-hippocampal networks (Staresina et al., 2015; Mitra et al., 2016; Chan et al., 2017), and Schwalm et al. found evidence for a resting-state fMRI connectivity network that was similar to the first of these. This suggests that these phenomena extend well beyond the cortex, with large-scale neural interactions at low frequency having an important role.

Of course, many questions remain in our quest to better understand and utilize resting-state fMRI connectivity networks. For example, how exactly do large-scale interactions within and between neural systems at rest give rise to distinct resting-state fMRI networks? How should we analyze and examine these networks to dissect their functional roles? The results of Schwalm et al., together with recent animal studies, signal that we are now entering an exciting phase in which the development of new strategies will allow us to explore the neural basis of resting-state fMRI connectivity networks even further.
